# The Influence of Roughness on the Protective Layer Formation Induced by Marine Microorganisms on 5083 Aluminum Alloy

**DOI:** 10.3390/ma18030708

**Published:** 2025-02-06

**Authors:** Julien Jaume, Marie-Line Délia, Régine Basséguy

**Affiliations:** Chemical Engineering Laboratory (LGC), Université de Toulouse, CNRS, INPT, UPS, 4 Allée E. Monso, 31432 Toulouse, France; julien.jaume@chimieparistech.psl.eu (J.J.); marieline.delia@toulouse-inp.fr (M.-L.D.)

**Keywords:** aluminum alloy, salt marsh microorganisms, surface roughness, microbially induced protection, marine corrosion, surface modification, biomineralization

## Abstract

This study investigates the formation of a protective layer on a 5083 aluminum alloy surface induced by microorganisms from salt marsh. The influence of the initial surface roughness was examined to identify optimal conditions for maximum coverage and thickness of the protective layer. As two opposing effects are suspected, where high surface roughness enhances bacterial adhesion but reduces the resistance to abiotic corrosion, various degrees of roughness were tested. Using electrochemical experiments (OCP measurement, 1/Rp determination, and pitting sensitivity), SEM/TEM observation and EDX characterization, a compromise was found on the initial roughness to obtain a thick protective layer through good bacterial adhesion while minimizing abiotic corrosion. The optimal roughness, achieved through 240-grit grinding, facilitates a uniform distribution of microorganisms and the development of a dense, evenly thick protective layer that significantly enhances the alloy’s resistance to pitting corrosion. The passivity domain doubled when comparing the electrochemical behavior of electrodes immersed in the presence of microbial activity to those immersed without it.

## 1. Introduction

Aluminum alloys are versatile materials used in a wide range of applications. Their high strength-to-weight ratio makes them especially practical when weight saving is required, such as in the aerospace, defense, construction, and transportation industries. Another advantage of these alloys is their resistance to corrosion [1]. In particular, 5xxx aluminum alloys (rich in magnesium) are often used in marine environments, which are particularly aggressive due to the high chloride concentration and the presence of microorganisms that can promote corrosion reactions [2,3,4,5]. In such an environment, even an alloy intrinsically resistant to corrosion will need to be protected [6,7,8].

Until recently, the most commonly used treatments to protect the aluminum alloys required the use of chromium VI [9,10,11], which is now prohibited by the European REACH (Registration, Evaluation, Authorization, and restriction of CHemicals) regulation [12]. These treatments coated the aluminum alloy with a protective layer that inhibits chloride adsorption [13], the first step in pitting corrosion process in seawater [14]. As the protective treatments currently available are not as effective, new solutions need to be developed to protect aluminum alloys in marine environments.

In this context, our previous publication studied the effect of salt marsh microorganisms on surface modification of 5083 aluminum alloy [15]. We concluded that salt marsh microorganisms, chosen for their capacity to form effective biocathodes and bioanodes [16,17], are involved in the formation of a homogeneous layer that protects against corrosion. It can be observed that in this study, the electrodes were cut from an aluminum alloy plate as it is used in marine industries, representative of real-world use. Other publications have shown the formation of a similar protective layer on 5083 aluminum alloy in several mediums: in seawater in Genoa [18,19,20], in an estuary environment in Lisboa [21] and in semi-field marine environment (reservoir filled with natural seawater, continuously renewed) [22]. Building on the achievements of our first study, it was next essential to understand the parameters that influence the formation of this protective layer, to further study the mechanisms involved. This required controlling the surface condition of the working electrode, which is crucial as a direct link has been established between the roughness of the metal and its susceptibility to corrosion [23]. Lower roughness is associated with (1) decreased reactivity of the surface due to the reduced surface exposed to the external environment and (2) better homogeneity of the natural oxide layer that insulates the aluminum against corrosion.

**Figure 1:**
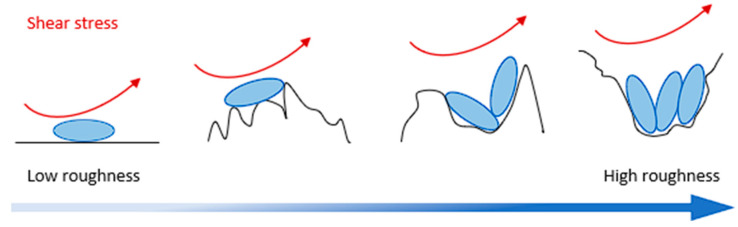
Influence of surface roughness on bacterial adhesion. Exposure to shear stresses from the medium is shown by the red arrows. The higher the roughness, the more shielded the bacteria are, which promotes their adhesion.

When microorganisms are involved, roughness also influences bacterial colonization and thus the development of a biofilm over the surface [24,25]. Indeed, the development of a biofilm first requires the adhesion of bacteria to the electrode surface. This adhesion step is hindered if the surface is too smooth as the bacteria have no shield against shear stresses caused by fluid dynamics in the medium (Figure 1). High surface roughness can support biofilm formation by preventing the bacteria from detaching, and the valleys caused by roughness are therefore preferential growth sites for biofilm [26].

Surface roughness is therefore associated with two antagonistic effects: on the one hand, resistance to abiotic corrosion increases as roughness decreases; but on the other hand, lower roughness negatively influences biofilm development by decreasing bacterial adhesion, consequently limiting the biofilm catalytic effect on corrosion reactions. To enhance the surface modifications presented in our previous publication [15], these effects had to be examined in relation to microorganism activity.

This new study aimed to identify the optimal electrode roughness that promotes biofilm development while minimizing sensitivity to abiotic corrosion. Several experiments were performed using different initial surface conditions, then the electrodes were analyzed using electrochemical methods and electronic microscopy observations. The resistance to pitting corrosion was evaluated for the electrodes with the optimized roughness to attest the protective properties of the layer formed.

## 2. Materials and Methods

### 2.1. Experiment Setup

**Figure 2:**
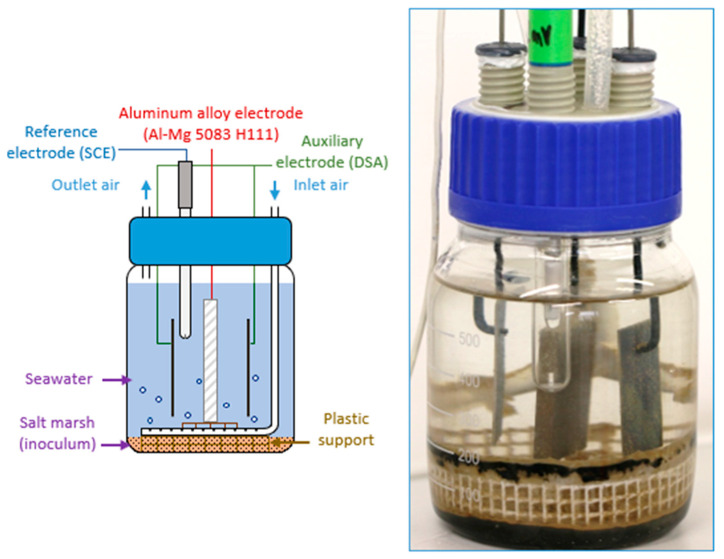
Experiment setup of the three-electrode system: scheme and photograph.

The reactor used was a 600 mL glass bottle containing the three different electrodes (Figure 2). The electrodes studied were made of aluminum alloy 5083 H111 (same origin and characteristics as in [15]) and measured 5 × 4 × 0.7 cm; their surface had been ground with silicon carbide (SiC) abrasive papers. The different surface conditions were obtained using, in turn, 80, 240, 400, and 800 grit abrasive papers to reach the desired level of grinding. For readability, the surface condition obtained after grinding using a xxx-grit abrasive paper is expressed as “ground to xxx” or “xxx-ground.” Two counter-electrodes (dimensionally stable anodes of iridium–tantalum supplied by MAGNETO, Schiedam, The Netherlands) were mounted on either side of the working electrode. The reference electrode was a saturated calomel electrode (SCE).

The electrodes were immersed for 21 days in a medium composed of natural seawater and salt marsh sediments, as sources of microorganisms, both collected at Gruissan (France). The experiments were conducted in duplicate, for each surface condition. The bacterial community of the salt marsh sediment was analyzed in other studies [16,17] and proved to remain constant in composition. The sediments are made up of two phases that separate after a few days’ storage: a solid phase (sludge) and a liquid phase. Our previous study [15] has shown that a mixture of 90% seawater, 5% of salt marsh sludge, and 5% salt marsh liquid is optimal for the surface changes we wanted to study here. A bubbling system connected to an air pump provided uniform aeration of the medium. The liquid part of the mixture was analyzed using ICP-AES with an Ultima Expert (Horiba) to measure the chemical concentration of main species (g/L): Cl^−^ (19.02), Na^+^ (11.61), SO_4_^2−^ (2.75), Mg^2+^ (1.43), K^+^ (0.52), Ca^+^ (0.43), HCO_3_^−^ (0.14). The pH was measured at 8.01 (24.7 °C) using a Meter lab PHM210 and the conductivity was measured at 5.12 S/m using a Sonde Eutech instrument Cont 6+.

### 2.2. Electrochemical Experiment

The electrodes were connected to a Biologic VMP2 potentiostat to perform electrochemical measurements. During the 21 days of immersion, the open circuit potential (OCP) was measured continuously, and a linear sweep voltammetry (LSV) was performed every 24 h. The LSV graphs, drawn at 0.167 mV/s from the OCP to −20 mV vs. OCP, were used to calculate the inverse of the polarization resistance 1/R_P_ that is proportional to i_corr_, using a linear regression close to the OCP value.

### 2.3. SEM/EDX Characterization

After 21 days of immersion, the Al–Mg electrodes were washed with distilled water and then prepared for surface or cross-section scanning electron microscope (SEM) observation. Cross-section samples were prepared putting half of the electrode (5 × 2 × 0.7 cm) into a cold mounting epoxy resin in order to observe the edge of the exposed surface. The embedded sample was then ground using SiC abrasives with several grain sizes up to 1000 grit. Samples were then polished using polishing cloth with diamond paste up to 1 µm grain size. Before a surface or cross-section observation, the sample was metallized with a 2 nm platinum layer (Leica EM ACE600 metallizer, Nanterre, France). SEM observations were conducted using a JSM 7100F (JEOL, Croissy-sur-Seine, France) scanning electron microscope. Images were acquired using a secondary electron detector. Energy Dispersive X-ray (EDX) characterization was performed using the ASDD X-Max 50 mm^2^ (Oxford, Les Ulis, France) EDX spectrometer to determine the elementary chemical composition.

### 2.4. TEM Analysis

Some cross-section samples were observed using a transmission electron microscope (TEM) JEM 2100F (JEOL) and EDX analysis was conducted with an energy dispersive X-ray spectrometer EDX (Brucker). The thin section (a few tens of nanometers) that was observed was machined using a focused ion beam with a FEI 600i (HELIOS, sold by Thermo Fisher Scientific, Illkrich, France) system.

### 2.5. Assessment of Protective Properties Through Pitting Corrosion Tests

After 21 days of immersion, some electrodes were tested to assess their resistance to pitting corrosion. Prior to each measurement, a 1 cm^2^ area was delimited on the electrode surface using an insulating varnish (Electrofuge 200-ND, CRC-KF, Argenteuil, France). The sample was then immersed in seawater for 24 h to ensure rehydration of the surface layer before conducting the tests. Stabilization of the potential was achieved by monitoring the OCP.

After this 24 h immersion period, resistance to pitting corrosion was evaluated using Cyclic Potentiodynamic Polarization (CPP). The potential was scanned from the OCP toward higher potentials at a rate of 0.167 mV/s. When the current reached the threshold of 100 µA, the E_pitting_ value was recorded, as it is indicative of the formation of a stable pit. The potential sweep direction was then reversed until it crosses the OCP curve, to determine the E_repassivation_ value.

Several passivation domains were then calculated: the total passivation domain (E_pitting_ − OCP), the perfect passivation domain (E_repassivation_ − OCP), where no pitting occurs, the imperfect passivation domain (E_pitting_ − E_repassivation_), where some metastable pits may appear.

### 2.6. Assessment of Surface Colonization by Microorganisms Using SEM/EDX

To enable observation of the surface colonization by microorganisms, some electrodes were treated as follows as soon as they were removed from the reactor. The biofilm was fixed by immersing the electrode for 20 min in a solution composed of: 2 volumes of a 4% glutaraldehyde solution (PanReac AppliChem, sold by Sigma Aldrich, Saint-Quentin-Fallavier, France), 1 volume of phosphate buffer (0.4 M and pH = 7.4; PanReac AppliChem), and 1 volume of deionized water (Elga Purelab Option-R, Veolia Water STI, Toulouse, France). The electrode was then washed by means of two successive 15 min immersions in a solution composed of: 2 volumes of sucrose solution (0.6 M; Thermo Fisher Scientific, Illkrich, France), 1 volume of phosphate buffer, and 1 volume of deionized water.

The last step consisted of biofilm dehydration. The electrodes were immersed for 5 min in a 50/50% deionized water/acetone (Thermo Fisher) mixture and then for 5 min in a 30/70% mixture, followed by immersion for 30 min in pure acetone. To complete the dehydration step, the electrode was immersed in a 50/50% acetone/HMDS (Sigma-Aldrich, Saint-Quentin-Fallavier, France) mixture for 30 min, and then in pure HMDS until evaporation.

Prior to the SEM observation, the pre-treated electrodes were metallized with a 2 nm platinum layer. Carbon EDX mapping was conducted to assess the distribution of organic matter linked to microbial activity, such as extracellular polymeric substance (EPS) and metabolites.

## 3. Results

### 3.1. Electrochemical Study

To study the influence of polishing on formation of the protective layer, the electrodes ground to different grades (80 to 800) were immersed for 21 days in reactors containing seawater with salt marsh sediment. This period of 21 days was chosen following previous works [15] where we clearly showed that the main protective layer formation phenomena were observed during the first twenty days. The open circuit potential (OCP) was monitored throughout the immersion for all the ground electrodes. Every 24 h, a linear sweep voltammetry (LSV) was also performed to calculate the value of the inverse of polarization (1/R_P_), proportional to the corrosion current i_corr_.

**Figure 3:**
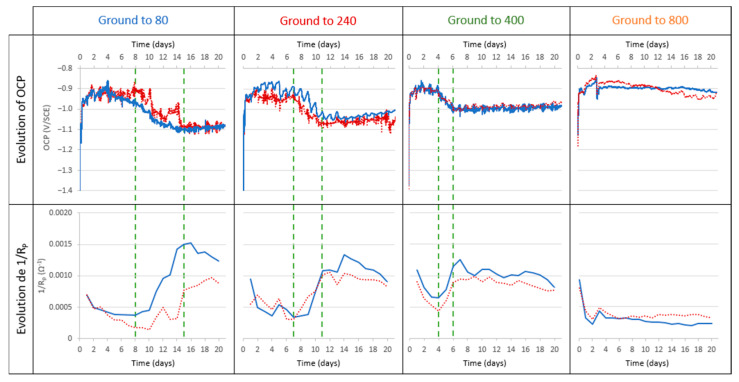
Electrochemical measurement results during the 21 days of immersion in biotic condition. The dotted green lines define the transition phase. The blue and red curves represent different duplicates for each condition.

The electrochemical measurement results, summarized in Figure 3, show similar OCP evolution for the electrodes ground to 80, 240, and 400. At the beginning of the immersion, the OCP stabilized around −0.9 V/SCE and remained stable for a specific period. Afterwards, the OCP decreases during a transition phase (bounded by two dotted lines in Figure 3) towards a value between −1.0 and −1.1 V/SCE. After this phase, the OCP remained stable until the end of the experiment. The final OCP values, as well as the different durations of each step, are summarized in Table 1. These values depend on the grinding of the aluminum alloy electrode. For electrodes ground to 800, there is no transition phase.

The variations of 1/R_P_ take place in conjunction with those of OCP. With the electrodes ground to 80, 240, and 400, the 1/R_P_ values remain quite low, around 5.10^−3^ Ω^−1^, and then increase simultaneously with the decrease in the OCP during the transition phase. After this phase, the OCP and 1/R_P_ stabilize concomitantly, however, with a trend of 1/R_P_ to decrease especially towards the end of the experiment. For the 800-ground electrode, the 1/R_P_ remains stable along with the OCP.

### 3.2. Photographs of Aluminum Alloy Electrodes

**Figure 4:**
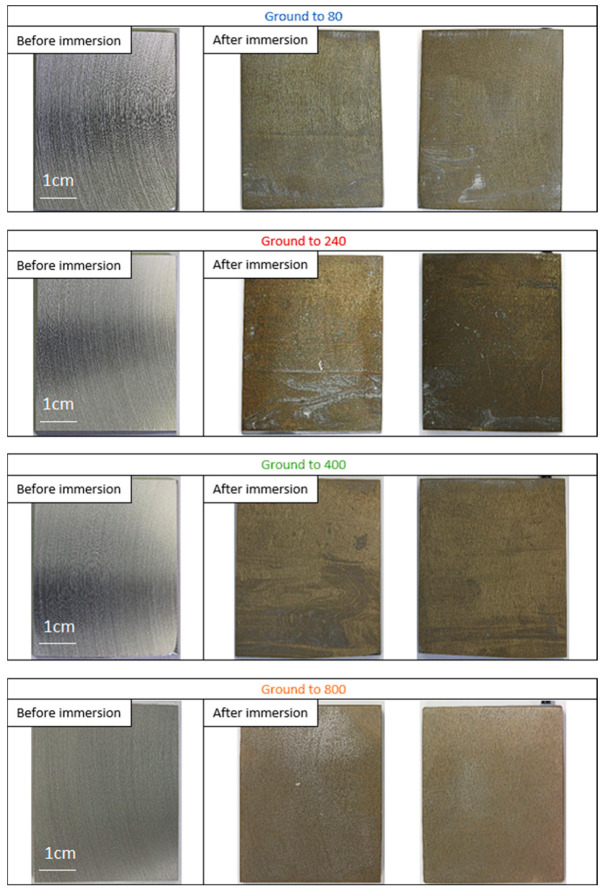
Photographs of electrodes ground to 80, 240, 400, and 800, before and after 21 days of immersion in biotic condition. Scratches are visible on samples before immersion. After immersion and cleaning of the surface, a black layer is observed on the surface with a more or less homogeneous distribution.

A picture of the electrodes was taken before and after the 21 days of immersion in seawater with salt marsh sediments (Figure 4).

Observation after the immersion period revealed the formation of a black layer, similar to the protective layer described in our previous study [15]. This layer appeared to cover the whole surface of the electrodes ground to 80, 240, and 400. The electrodes ground to 240 and 400 had a similar surface after immersion, with the scratches from the initial polishing no longer visible on them, whereas these scratches did remain visible on the electrodes ground to 80. Unlike the electrodes ground to 80, 240, and 400, the one ground to 800 appeared not to be completely covered by the layer.

### 3.3. Surface SEM/EDX Characterization

**Figure 5:**
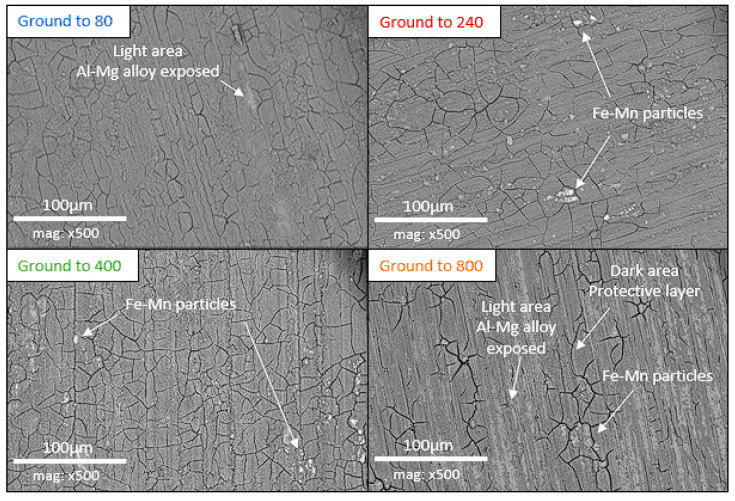
SEM observation of the aluminum alloy surface for different grinding grades after 21 days of immersion in biotic condition.

**Figure 6:**
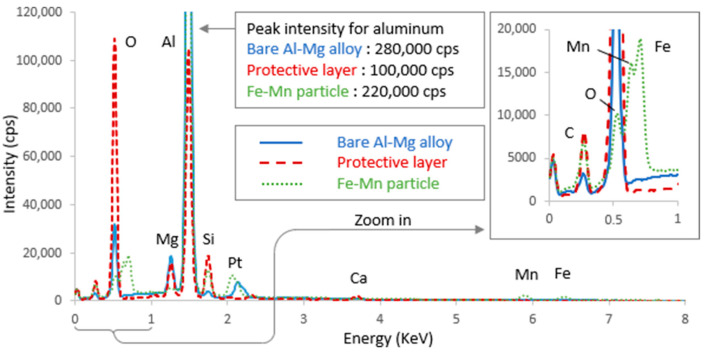
Comparison of EDX analysis of a dark area (with the layer), a light area (bare Al-Mg alloy), and a white spot (Fe-Mn particle) observed on the SEM image of aluminum alloy ground to 800, after 21 days of immersion in biotic condition.

After the 21 days of immersion, SEM observations of the surface of the aluminum alloy samples revealed that they were all covered with a cracked layer (Figure 5). These cracks probably resulted from shrinkage of the layer caused by its dehydration after emersion. This effect may be accentuated by the vacuum applied to the samples observed with the SEM. According to the EDX analysis (Figure 6), this cracked layer is mainly composed of oxygen and aluminum, and small amounts of silicon, magnesium, and calcium. This composition matches that of the protective layer described with more detail in the previous study [15]. The white spots visible on the images correspond to iron and manganese particles. On the electrodes ground to 240 and 400, the layer appears to cover the whole surface homogenously.

On the electrode ground to 80, there are a few light areas (lighter in color than the layer), which appear not to be covered. The electrode ground to 800 presents a larger number of light areas. EDX analysis revealed that the light areas are mostly composed of aluminum, oxygen, and magnesium, confirming that they are not covered by the layer. We can conclude that the layer did not develop uniformly on the surface of electrodes ground to 800. No light areas are observed on the electrodes ground to 240 and 400, confirming that their surface was covered wholly and uniformly.

To sum up, observation of the electrodes’ surfaces revealed a difference in coverage by the cracked layer, in line with the macroscopic observations described in the previous section. Grinding too smooth a grade, such as 800, hinders the development of a uniform layer, but grinding above a certain roughness, such as 80, may have a similar effect, albeit less marked. The surface conditions obtained with intermediate grinding, to 240 and 400, promoted uniform development of a layer over the entire surface.

### 3.4. Cross-Section SEM/EDX Characterization and Thickness of the Layer

**Figure 7:**
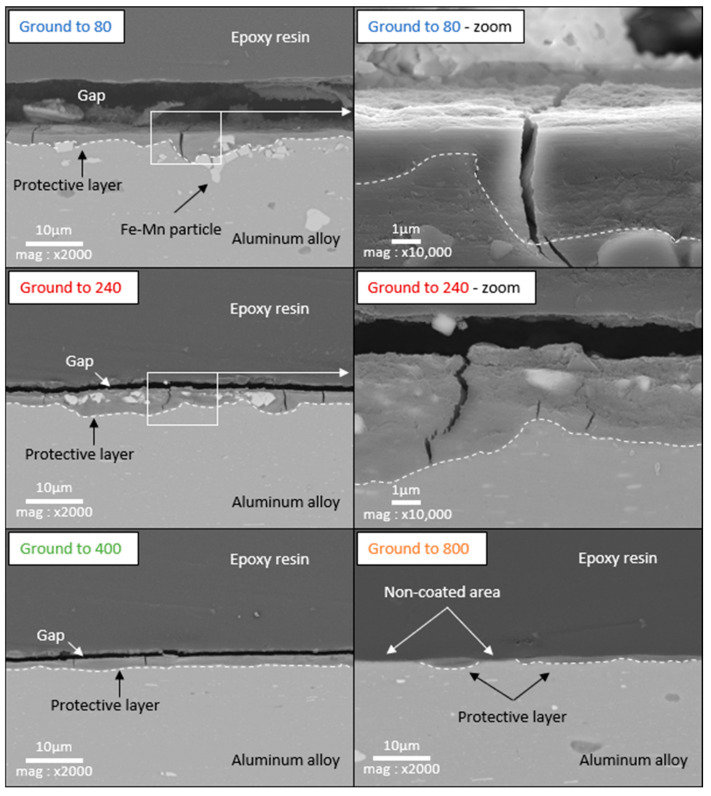
SEM cross-sectional observation (backscattered electrons) of aluminum alloy samples ground to 80, 240, 400, and 800, after 21 days of immersion in biotic condition.

The electrodes were also observed in cross-section SEM (Figure 7). At the top of most images, the epoxy resin can be seen over the layer that has developed directly against the aluminum alloy. However, the resin appears not to adhere perfectly, resulting in gaps between the resin and the layer. EDX analysis (not shown here for brevity) indicates that this layer corresponds to the layer observed by SEM on the surface. This layer has a variable thickness but covers the majority of the aluminum alloy on the electrodes ground to 80, 240, and 400. On the sample ground to 800, we notice that some areas appear not to be covered by the layer, corresponding to the light areas detected in the SEM observations of the surface. Cross-sectional observations also reveal several cracks in the layer (zoomed view). The cracks appear clean, supporting the hypothesis that they formed after emersion. There are no signs of corrosion at the base of the cracks, which would have been expected if the cracking had occurred during immersion, as this would have exposed the aluminum alloy to the aggressive environment. The cross-section observation also shows that the layer seems to be more developed around Fe–Mn particles.

**Figure 8:**
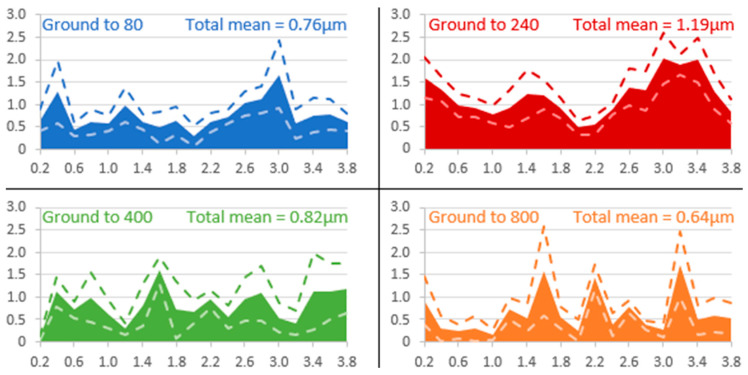
Distribution of average layer thicknesses measured along each ground sample, along with the total average of the measurements. The dashed curves represent the standard deviation for each average. The *x*-axis is the distance from an edge of the plate (cm) and the *y*-axis is the average thickness (µm).

From the cross-sectional observations, the thickness of the layers was measured over a length of 4 cm of the sample using SEM images (mag ×5000, covering a width of 24 µm) captured every 0.2 cm. The edges of the sample were voluntarily excluded, so 19 electronic images were captured per sample. For each image, 10 measurements were taken, at regular intervals (every 2 µm). Figure 8 represents the distribution of the average layer thickness, as well as its standard deviation, along the length of the sample for each grinding grade. Each point in these graphs corresponds to an average of 10 measurements made on an SEM image. The standard deviation of these averages is displayed as two dotted lines on the graphs. The thickness of the layer is heterogeneous regardless of the grinding grade, showing significant disparity in its averages, with a high standard deviation. The maximum standard deviation in layer thickness on the electrodes ground to 80, 240, 400, and 800 is 0.7 µm, 0.5 µm, 0.85 µm, and 1 µm, respectively.

### 3.5. TEM Observation of the Layer

**Figure 9:**
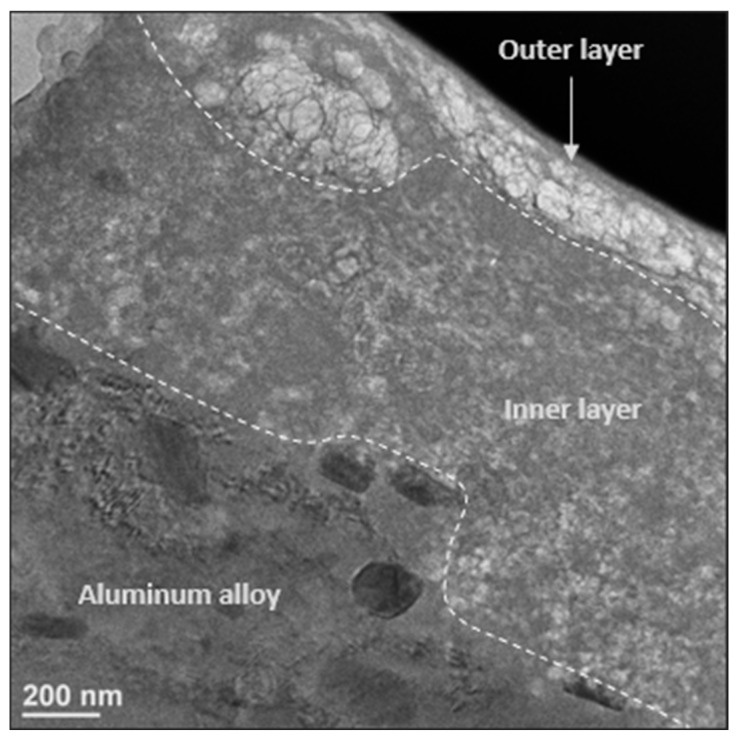
Bright field TEM image of a cross-sectional view of the 240-ground aluminum alloy plate, after immersion for 21 days in biotic condition.

A cross-section of the sample ground to 240, made using focused ion beam machining, was observed by TEM (Figure 9). The layer can be seen as being composed of two layers: a dense inner layer over the aluminum alloy, over which there is a more porous outer layer.

A similar bilayer structure was previously observed by TEM and analyzed using EDX for a non-grinded electrode immersed in the same medium for 50 days [15]. The inner layer of the electrode immersed for 21 days, and 50 days have an equivalent thickness (≈800 µm), but the outer layer of the electrode immersed for 21 days is thinner (≈200 µm) than for 50 days (≈500 µm).

### 3.6. Assesment of the Protective Properties of the Formed Layer

**Figure 10:**
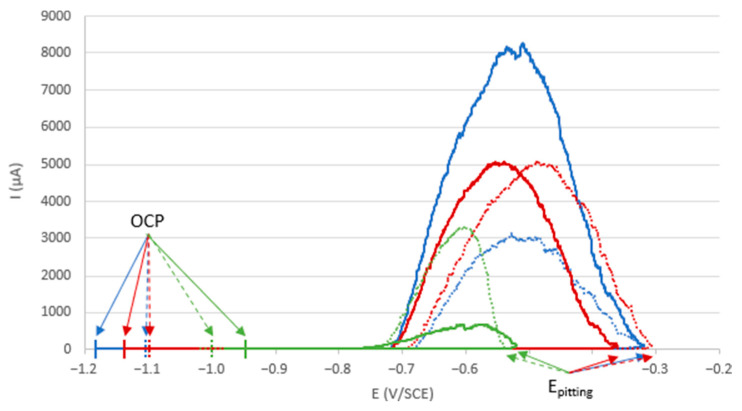
Pitting curves for the 240-ground aluminum alloy electrode drawn in seawater after 24 h of immersion. Two samples were immersed for 21 days under biotic conditions (blue and red curves), while one sample was immersed under abiotic conditions (green curve). The solid and dotted lines represent two measurements taken from the same sample.

To assess resistance to pitting corrosion, Cyclic Potentiodynamic Polarization (CPP) measurements were performed on electrodes after 24 h of immersion in the reactor containing seawater. Three 240-ground samples were tested: two samples after immersion for 21 days under biotic conditions and one sample after immersion for 21 days under abiotic conditions (same medium but with autoclaved salt marsh sediments). Two 1 cm^2^ zones (delimited using an insulating varnish, see Section 2.5) were analyzed for each sample (Figure 10). When comparing the experiments conducted under biotic conditions to those under abiotic conditions, the OCP were lower, while the pitting potential was significantly higher. The repassivation potentials were similar.

The data extracted from the pitting curves are summarized in Table 2. The total passivity domains, as well as the perfect and imperfect passivity domains, were calculated (see details in Section 2.5). Under biotic conditions, these domains were twice as large as those under abiotic conditions, demonstrating the superior pitting resistance of the samples immersed in the presence of bacteria.

### 3.7. Assessment of Surface Colonization by Microorganisms Using SEM/EDX

**Figure 11:**
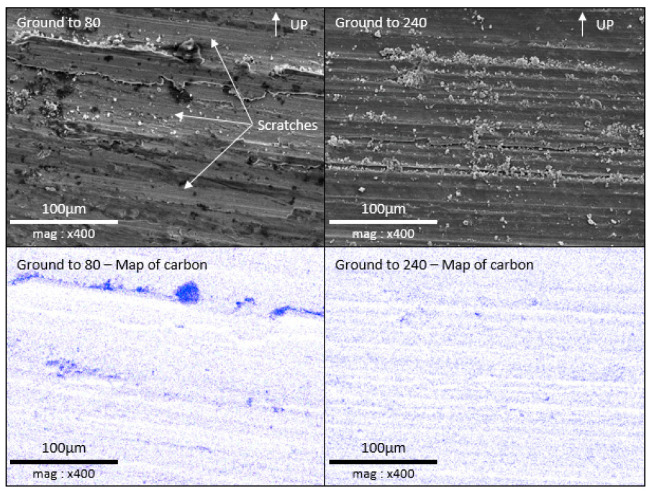
SEM observation and EDX mapping of carbon for the surface of 80- and 240-ground electrodes, after treatment for biofilm fixation. A white “UP” arrow indicates the top of the electrode when it was submerged in biotic condition.

Using the same experiment setup described for 21-day immersion, 80- and 240-ground electrodes were immersed for 24 h. The electrodes were held upright in the reactor and the scratches from the grinding process were horizontal. After immersion, these electrodes were treated to fix the microorganisms/biofilm on the surface for SEM observation. Figure 11 shows the SEM observation of the electrode surface and the carbon mapping performed by EDX analysis. The surface of the 80-ground electrode presents deep and wide scratches (valleys). The EDX analysis shows a highly heterogeneous distribution of the carbon, which is mainly localized at the bottom of the valleys. The 240-ground electrode presents a surface with shallower and more numerous scratches. This surface is easily seen with carbon mapping: in this case, the carbon is more evenly distributed than on the 80-ground electrode.

**Figure 12:**
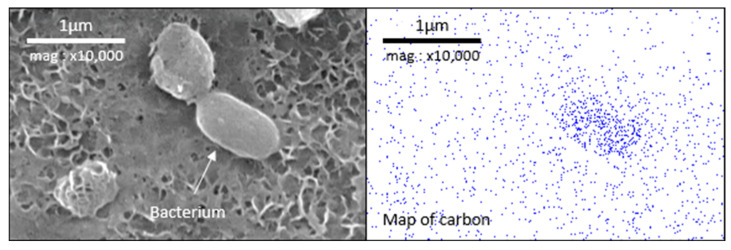
SEM observation and EDX mapping of carbon for the surface of a 240-ground electrode and zoomed view of a bacterium.

Figure 12 shows a bacterium with the associated carbon mapping. This shows a strong correlation between high carbon concentration and the presence of microorganisms. According to EDX analysis, the white deposits on the electrode surface are mainly composed of oxygen and silicon (data not shown for brevity). It is assumed that these deposits are not of bacterial origin; rather, they are probably mineral particles from the salt marsh that became trapped in the surface asperities because the electrode was not rinsed before the biofilm fixation treatment. We were therefore able to use carbon mapping to map the presence of microorganisms organized in biofilm over a broad surface, to assess the influence of the grinding process on biofilm development. For the 80-ground electrode, a biofilm developed mainly at the bottom of the scratches, where bacteria were held in place by the shape of the valley, which shields them against shear forces. This results in a discontinuous accumulation of organic matter on the surface. On the 240-ground electrode, similarly to the 80-ground electrode, a biofilm developed at the bottom of the scratches; however, because these scratches are shallow, the biofilm has been able to partially cover the areas between the scratches. Biofilm development is more uniform and covers more surface area with 240-grit polishing than with 80-grit polishing.

## 4. Discussion

Overall, our observations indicate a clear correlation between initial roughness, and the characteristics of the layer formed (coverage and thickness of the protective layer):

- Low roughness, ground to 800: photos taken after the immersion phase show that the 800-ground electrode presents uncoated areas (Figure 4). Indeed, after immersion, the surface is not fully covered by the layer, according to SEM observations of the surface (Figure 5) and cross-section (Figure 7). Moreover, the layer has a significantly lower average thickness than for electrodes ground to other grades (Figure 8). The low roughness obtained with the 800 grinding is therefore unfavorable for the formation of an evenly distributed protective layer.
- High roughness, ground to 80: SEM observation of the surface reveals that a few areas are not covered by the layer, but these areas are much less numerous than with the 800 grinding. The average thickness is significantly lower than with the 240 grinding. Therefore, the 80 grinding does not produce the optimum roughness.
- Intermediate roughness, ground to 240 and 400: according to photos and SEM observations, electrodes ground to these grades have a layer that covers their entire surface, contrary to the 80- and 800-ground electrodes. However, the thickness of the layer obtained on the 240-ground electrode is significantly higher and more homogeneously distributed (lower standard deviation) than the layer obtained on the 400-ground electrode. Moreover, the thickness of this layer is higher with the 240-ground electrode than any layer obtained with any other grinding process. Therefore, grinding to 240 provides the best initial surface condition to promote the development of the layer on the surface of the aluminum alloy. The protective nature of the layer was clearly demonstrated (Figure 10) by a twofold increase in the passive domain (both perfect and imperfect). The layer is thought to act as an anti-corrosion coating by limiting the penetration of chloride ions to the substrate below. A layer that is homogeneous in thickness and composition will be more effective in protecting the substrate underneath, as observed with the 240 ground electrodes.

### 4.1. Influence of Surface Roughnees on Electrochemical Behavior

**Figure 13:**
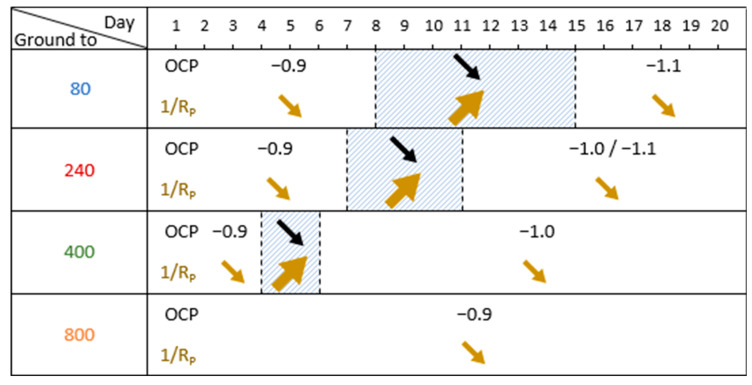
Evolution of OCP and 1/R_P_ during immersion in biotic conditions, according to the different grinding processes. The arrows indicate the direction of variation, the intensity of which is represented by the thickness of the arrow. The hatched section shows the transition zone.

The electrochemical study highlights that the measured OCP, and the calculated 1/R_P_, evolve differently during immersion according to the grinding process. The evolution of these parameters is summarized for the different experiments according to the grinding grade (Figure 13). This shows a correlation between this evolution and the initial surface condition. For the electrodes ground to 80, 240, and 400, we can define a transition zone for which we observe a decrease in the OCP (from −0.9 V/SCE to about −1.1 V/SCE) and an increase of 1/R_P_. The finer the grinding, the earlier this transition phase occurs, and the shorter its duration. The 800-ground electrodes differ from the others in that they present no transition zone: the OCP remains at a steady value of around −0.9 V/SCE for the 21 days of immersion, and 1/R_P_ remains at a low value and decreases slightly. The 800-ground electrodes are the only ones that are not completely covered by the layer. This transition phase is therefore correlated with the formation of the layer and full coverage of the electrode surface.

### 4.2. Role of Microorganisms in Protective Layer Formation: Biotic vs. Abiotic Conditions

**Figure 14:**
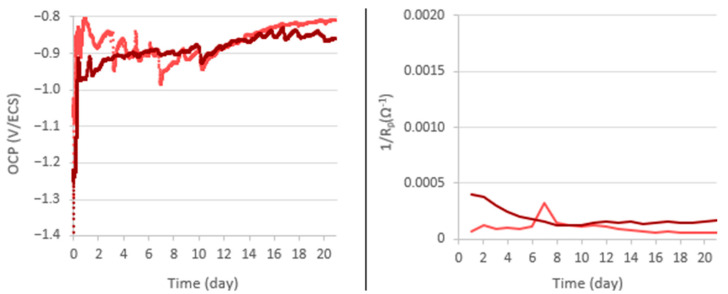
Evolution of OCP and 1/R_P_ for an experiment with an electrode ground to 240, in abiotic conditions. Each color corresponds to a replicate.

To complete the study and establish the influence of microorganisms, abiotic reference experiments were also conducted. Electrodes ground to 240 were immersed during 21 days in a medium with autoclaved salt marsh sediments. The electrochemical results are presented in Figure 14. During the abiotic immersion, no transition zone emerged; the OCP and the 1/R_P_ remained stable. These results differ from the results obtained under biotic conditions for the same grinding process. The OCP in abiotic conditions remained stable and above −0.9 V/SCE, which is a higher value than for the electrodes exposed to microorganisms. For the electrodes immersed in abiotic conditions, SEM observations (surface and cross-section) showed no protective layer, confirming that the transition zone observed in the biotic conditions and the subsequent drop in OCP are strongly correlated to the formation of a layer over the whole surface.

We also notice that the 1/R_P_ values obtained during experiments in abiotic conditions are lower (<2.10^−4^ Ω^−1^) than those obtained in experiments under biotic conditions (Figure 3). An incorrect interpretation of these results would be to conclude that, in abiotic conditions, electrodes are more protected because there are fewer electrons exchanged. Indeed, electron exchange seems to be necessary for the formation of the protective layer. In abiotic conditions, there is no significant corrosion of the electrode but there is also no formation of the protective layer. This layer can be considered as a protective coating during exposure to corrosive media (as observed in pitting tests). The comparison of 1/R_P_ values for 240-ground electrodes in abiotic conditions with those for 800-ground electrodes in biotic conditions (~3 × 10^−4^ Ω^−1^) reveals an interesting observation: the 1/R_P_ values for 240-ground electrodes are lower. Normally, under purely abiotic conditions, 1/R_P_ values for 240-ground electrodes would be higher than those for 800-ground ones due to their increased surface roughness. This observation indicates that the activity of even a limited number of microorganisms significantly influences the development of the protective layer. However, to form a thick, evenly distributed layer that effectively shields the entire surface, a higher level of microbial colonization is required.

### 4.3. Mechanisms of Dissolution-Precipitation in Layer Formation

Indeed, in biotic conditions, the marine aerobic biofilm that develops on the surface of the electrodes (initial OCP around −0.9 V/ECS) is known to catalyze the reduction of oxygen [16]. This results in an increased pH near the surface, further destabilizing the native passive oxide layer (together with chloride) and promoting dissolution of the aluminum alloy (increase of 1/Rp and decrease in OCP). Subsequently, the elements present in the surrounding environment, including aluminum alloy that has been dissolved and hydroxide ions, can precipitate as protective oxides. Therefore, the high level of electron exchange is related to the reduction of oxygen paired with the dissolution of aluminum and therefore relates to the formation of the layer. This phenomenon of dissolution–precipitation also seems to be exacerbated at the Fe–Mn inclusions because they are efficient cathodic sites, meaning that the oxygen reduction is catalyzed at their location. The layer around these inclusions appears effectively thicker (Figure 7, Ground to 240—Zoom). After the transition phase, the electrodes reach a stable OCP value of −1.0/−1.1 V/SCE while the protective and insulating layer covers the entire surface, leading to the electron exchange decreases along with the 1/R_P_ value at the end of the experiment.

### 4.4. Bacterial Adhesion and Surface Roughness

**Figure 15:**
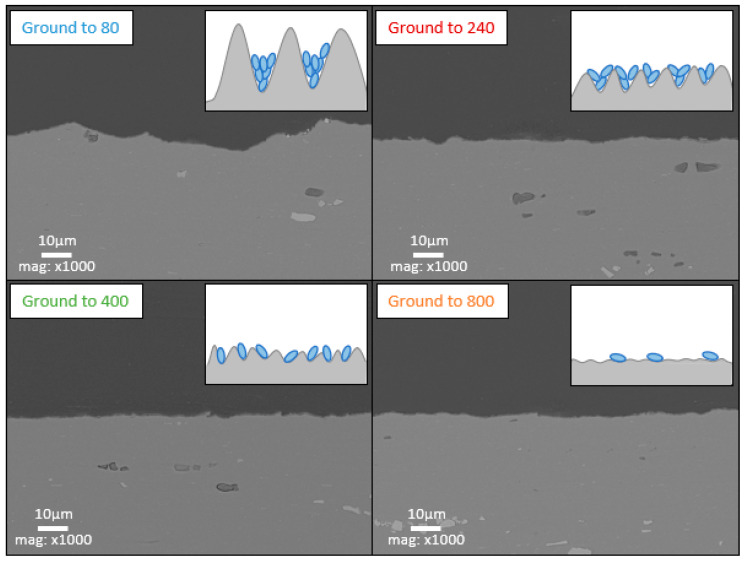
SEM (backscattered electrons) cross-section observations of an Al–Mg electrode for different grinding before immersion. For each grinding, a drawing represents the bacterial colonization (blue).

Comparing the electrochemical results indicates that protective layer formation is linked to bacterial colonization, which is influenced by the initial roughness of the surface. To explain these results, Figure 15 combines the cross-sectional SEM observations of the ground Al–Mg electrodes prior to immersion with drawings representing the associated bacterial colonization. For rough grinding, such as 80-grit, the scratches are deep, creating a significant difference in height between the peaks and valleys. This allows bacteria (depicted in blue in the figure) to settle and be well protected from shear stresses at the bottoms of the scratches, though their distribution remains discontinuous. In contrast, finer grinding, such as 800-grit, results in more scratches over the same area, with smaller height differences between the peaks and valleys. This diminishes the active surface and provides less effective protection against the shear stresses for the bacteria at the bottom of the shallower scratches.

Consequently, bacteria adhere more easily in the deeper hollows caused by high roughness, as achieved with 80 grinding. However, roughnesses close to the size of bacteria are the most optimized for bacterial adhesion, providing both protection from shear stress and a larger surface area to colonize [27,28]. If the hollows are too deep, the bacteria are protected from shear forces, but the steeper peaks will require more time to colonize.

In our conditions, it is difficult to locate the microorganisms because the consequence of their development is the formation of the layer. After the 21 days of immersion, the microorganisms’ distribution locations are hidden. To illustrate this point, we replicated the experiment with a 24 h immersion time. The microorganisms could not be observed by epifluorescence due to the autofluorescence of the material; thus, in order to determine the distribution of microorganisms, they were fixed and then observed by SEM/EDX (Figure 11). Carbon mapping, which correlates with the presence of microorganisms on the surface, confirms that biofilm preferentially develops in valleys where the microorganisms are protected from shear forces. In addition, the vertical position of the electrode means that the upward-facing surface of the bumps (which protrude horizontally) more effectively retains microorganisms transported by the moving medium. This is clearly visible on the surface of the 80-ground electrodes, where rough scratches are more easily colonized on their upward-facing surface than on their downward-facing surface. It is also noticeable that, on 80-ground electrodes, less biofilm forms between these preferential zones. In the case of finer grinding, such as 240, the scratches are more numerous and shallower. As a result, there are more zones favoring the colonization then the development of the biofilm that can spread more easily between preferential zones. Biofilm is therefore more evenly distributed.

Furthermore, a study on *Pseudomonas aeruginosa* adhesion to steel surfaces [29] provides relevant insights. In this publication, greater bacterial coverage was observed on 500-ground coupons (images E and F of Figure 3 in [29]) compared to 80-ground coupons (images G and H) under the same exposure conditions. On the 500-ground surface, bacteria observed through epifluorescence microscopy were evenly distributed across the entire surface, effectively concealing the numerous scratches caused by the grinding process. These scratches, having sufficient depth, facilitate bacterial colonization. The low height difference between the hollows and peaks allows biofilm to form uniformly across the scratches caused by polishing. In contrast, for the 80-ground surface observed via epifluorescence microscopy, the polishing stripes are significantly more visible compared to the 500-ground surface. In this case, bacterial colonization occurred predominantly in the hollows of the stripes, leaving more black zones (areas without bacteria) due to the greater height difference between the hollows and peaks. There are interesting parallels between the findings in this publication and our study. The biofilm distribution and mechanisms described help to explain the longer time required to achieve a homogeneous bacterial layer on the 80-ground surface compared to the 240 and 400-ground surfaces. For the 80-ground surface, which exhibits the highest roughness, the transition phase occurs later and extends over a longer period. This delay arises because bacteria preferentially colonize the hollows, requiring more time to spread to the peaks.

### 4.5. Optimal Grinding Conditions for Protective Layer Development

The duration and the time before the transition phase occurs increase as roughness decreases. The 400 grinding might, therefore, appear to be the most efficient, as this phase begins the fastest and requires the least time. However, the biomineralized layer produced by microorganisms is thinner compared to the 240 grinding. It is possible that the biofilm rapidly covers the electrode due to the shallow valleys associated with low roughness; however, the number of bacteria is lower. This leads to the quick formation of a uniform but relatively thin layer. While the 400 grinding provides more sites favorable for biofilm growth (valleys) than the 240 grinding, this comes at the expense of bacterial density, resulting in lower overall bacterial activity.

The roughness of the 800-ground surface is insufficient, explaining the low quantity of microorganisms present on its surface. The low roughness of the surface does not offer adequate protection of the bacteria that try to settle on it, and only a few zones are favorable for bacterial adhesion. Indeed, the contact surface with the metal is reduced [30]. As a result, there are not enough microorganisms to ensure the formation of a layer covering the entire surface before the end of the experiment. The transition zone of OCP and the increase 1/R_P_ are therefore not observed.

## 5. Conclusions

The experiments presented in this study demonstrate the influence of surface roughness on the formation of a protective layer induced by microorganisms from salt marsh sediments on 5083 aluminum alloy. SEM observations revealed the extent of the layer’s coverage on the alloy surface and allowed the measurement of its thickness for each distinct grinding process. Electrochemical results highlighted a transition zone, also influenced by surface roughness, during which significant electron exchange occurs, leading to the development of a protective layer.

An analysis of all results led to the formulation of a hypothesis explaining how surface roughness affects layer formation through the adhesion of microorganisms to the aluminum alloy. Our findings suggest that 240-grit grinding offers the optimal balance between roughness (providing a favorable number and depth of adhesion sites) and the size of the microorganisms, facilitating uniform colonization across the electrode’s surface. This even distribution of microorganisms promotes homogeneous growth of the protective layer. Understanding these phenomena provides valuable insights that could promote the development of environmentally friendly processes for producing effective anti-corrosion coatings.

## Figures and Tables

**Table 1:** Summary of the day number and duration of the transition phase, as well as the OCP after this phase.

Electrode Ground to	Transition Phase		OCP After Transition Phase (V/SCE)
	Start at	Duration (day)	
80	8th day	7	−1.1
240	7th day	4	−1.1 to −1.0
400	4th day	2	−1.0

**Table 2:** Main CPP parameters extracted and calculated passivity domains for the 240-ground aluminum alloy plate.

Sample	OCP (V/SCE)	E_pitting_ (V/SCE)	E_repassivation_ (V/SCE)	Passivity (V)	Perfect Passivity (V)	Imperfect Passivity (V)
Sample 1	−1.15 ± 0.03	−0.31 ± 0.00	−0.72 ± 0.04	0.84 ± 0.03	0.43 ± 0.07	0.41 ± 0.04
Sample 2	−1.13 ± 0.01	−0.33 ± 0.03	−0.73 ± 0.03	0.80 ± 0.04	0.40 ± 0.04	0.40 ± 0.06
Sample abiotic condition	−0.99 ± 0.04	−0.53 ± 0.01	−0.77 ± 0.04	0.46 ± 0.04	0.22 ± 0.08	0.24 ± 0.05

## Data Availability

The original contributions presented in this study are included in the article. Further inquiries can be directed to the corresponding author.
